# Core-Shell Silver/Polymeric Nanoparticles-Based Combinatorial Therapy against Breast Cancer *In-vitro*

**DOI:** 10.1038/srep30729

**Published:** 2016-08-05

**Authors:** Nancy M. Elbaz, Laila Ziko, Rania Siam, Wael Mamdouh

**Affiliations:** 1 Yousef Jameel Science and Technology Research Center (YJSTRC), School of Sciences and Engineering, American University in Cairo, 11835, Egypt; 2 Department of Chemistry, School of Sciences and Engineering, American University in Cairo, 11835, Egypt; 3 Department of Biology and Biotechnology Graduate Program, School of Sciences and Engineering, American University in Cairo, 11835, Egypt

## Abstract

The current study aimed at preparing AgNPs and three different core-shell silver/polymeric NPs composed of Ag core and three different polymeric shells: polyvinyl alcohol (PVA), polyethylene glycol (PEG) and polyvinylpyrrolidone (PVP). Thereafter, the core/shell NPs were loaded with a chemotherapeutic agent doxorubicin (DOX). Finally, the cytotoxic effects of the different core-shell Ag/polymeric NPs-based combinatorial therapeutics were tested *in-vitro* against breast cancer (MCF-7) and human fibroblast (1BR hTERT) cell lines. AgNPs, Ag/PVA and Ag/PVP NPs were more cytotoxic to MCF-7 cells than normal fibroblasts, as well as DOX-Ag, DOX-Ag/PVA, DOX-Ag/PEG and DOX-Ag/PVP nanocarriers (NCs). Notably, low dosage of core-shell DOX-loaded Ag/polymeric nanocarriers (NCs) exhibited a synergic anticancer activity, with DOX-Ag/PVP being the most cytotoxic. We believe that the prepared NPs-based combinatorial therapy showed a significant enhanced cytotoxic effect against breast cancer cells. Future studies on NPs-based combinatorial therapy may aid in formulating a novel and more effective cancer therapeutics.

Breast cancer is the most common type of cancer among women worldwide^1^,^2^. DOX is an FDA-approved chemotherapeutic agent frequently used in the treatment of various cancers including breast cancer. DOX is an anthracycline drug that inhibits topoisomerase-II-mediated DNA repair and leads to cell apoptosis^3^. Despite the potent anticancer action of DOX, it mediates cardiotoxicity. It was reported that DOX cumulative dose was the only confirmed risk factor for DOX-mediated cardiotoxicity. Furthermore, drug-induced cancer resistance is another obstacle that limits DOX clinical use^4^. A current approach used to overcome the resistance problem is the utilization of two or more chemotherapeutics^5^. Despite the synergism mediated by such combination chemotherapies, clinical studies showed that pharmacokinetic interactions of such combination chemotherapies induced severe systemic side effects such as cardiotoxicity and bone marrow suppression^5^,^6^,^7^. Consequently, there is an urgent need for the development of novel strategies to treat cancer. One strategy is to modify a well-studied chemotherapeutic agent, e.g. DOX- that would ideally: (i) target and kill cancer cells selectively, (ii) have an improved efficacy/toxicity balance, (iii) have an enhanced therapeutic index and (iv) have an improved pharmacokinetics profile; when compared to the original non-modified drug.

Nanotherapeutics usage in drug delivery applications has recently increased because of their desirable therapeutic characteristics e.g. prolonged systemic circulation and targeted drug delivery^7^,^8^,^9^. The previous properties are particularly advantageous for cancer therapeutics because they would result in an improved chemotherapeutic efficacy and would minimize the systemic toxicity^8^,^9^,^10^. The advent of nanotechnology allowed the emergence of several formulation techniques of NPs-based combinatorial therapy. These techniques include multiple chemotherapeutics using a single NC in order to overcome the drug-induced cancer resistance^11^. Recently, another approach similarly employed NPs-based combinatorial therapy, utilizing NPs with anti-cancer activity in combination with a chemotherapeutic agent. Ostad *et al.* used this approach and reported that administrating a low dose of tamoxifen following AgNPs to breast cancer cells and tamoxifen-resistant cancer cells induced a synergistic anticancer effect against both cancer cell lines^11^. Several studies reported that the cytotoxicity of core-shell Ag/PVP NPs was attributed to their ability to bypass the cell membrane via endocytosis and to localize into the lysosomes. Here, the NPs are exposed to an acidic environment that triggers the release of silver ions, eventually inducing the generation of reactive oxygen species (ROS). The generated ROS and AgNPs escape from the lysosomes and then they disrupt the mitochondria. This event leads to the generation of more ROS, ultimately leading to DNA damage and cell death^12^,^13^,^14^,^15^,^16^,^17^. AgNPs were reported to possess potent anti-angiogenic effects via inhibition of vascular endothelial growth factor (VEGF)-induced angiogenesis both *in-vitro* and *in-vivo*^18^. In addition, Kwon *et al.* showed that surfactant-coated AgNPs are hemo-compatible with human erythrocytes, and their conclusion supports the idea of intravenous administration of AgNPs as well as their potential use for cancer therapy^16^,^19^.

This study aimed at formulating combinatorial nanotherapeutics by conjugating DOX onto core-shell Ag/polymeric NPs. The NPs were composed of Ag as the core and three FDA-approved polymers (PVA, PEG and PVP) as the shell (Fig. 1). The drug loading and *in-vitro* drug release of DOX-NCs were monitored and their cytotoxic effects on MCF-7 cells and 1BR hTERT cells were assessed. Our results demonstrate that core-shell Ag/polymeric NPs and DOX-NCs had higher efficacy in killing MCF-7 cells -in comparison to their unloaded counterparts. Our results imply the high potential of the use of the synthesized DOX-loaded Ag/polymer NCs as nanotherapeutics for breast cancer treatment in the future.

## Results

### Synthesis and Characterization of AgNPs and core-shell NPs

**Figure 2:**
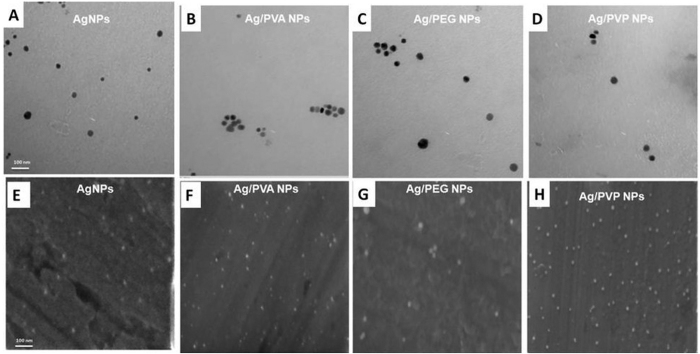
(**A**–**D**): Representative TEM images and (**E**–**H**) SEM images of AgNPs, and core-shell Ag/polymeric NPs.

**Figure 3:**
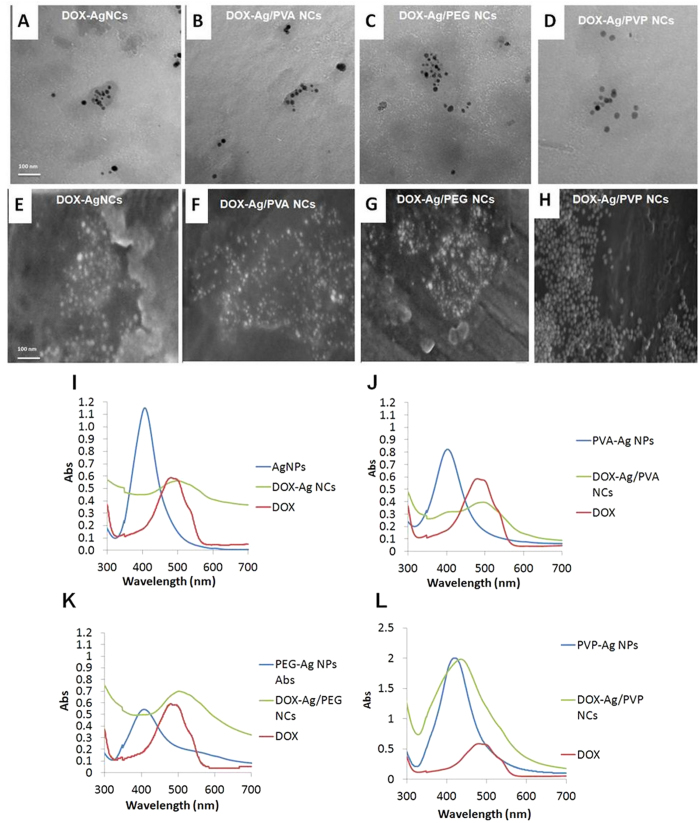
TEM and SEM images of DOX-Ag NCs and DOX-Ag/Polymeric NCs, respectively. UV–Vis spectra of DOX-AgNCs (**I**) and DOX-core-shell Ag/Polymeric (PVA, PEG, and PVP) NCs (**J**–**L**). Each UV–Vis spectrum is a comparison between UV–Vis spectra of NPs, DOX, and DOX-NCs.

The size and morphology of the prepared AgNPs and core-shell Ag/polymeric NPs were characterized by TEM, SEM, and UV-visible spectroscopy. TEM and SEM images showed that the prepared AgNPs and core-shell Ag/polymeric NPs were spherical, mono-dispersed, and well-dispersed (Fig. 2). The size and zeta potential of AgNPs and core-shell Ag/polymeric (PVA, PEG, and PVP) NPs (Table 1) were measured using the Zetasizer and were found to be in the range of 15–28 nm. The zeta potential values of AgNPs, Ag/PVA NPs, Ag/PEG NPs and Ag/PVP NPs were −12.43 ± 1.20 mV, −0.30 ± 0.50 mV, −2.35 ± 1.76 mV and −12.4 ± 1.20 mV respectively. Although the zeta potential of core-shell NPs demonstrated relatively low values, these NPs are stable due to the presence of a large molecular weight stabilizer that acts mainly by steric stabilization. This is based on the fact that the adsorbed polymer layer shifts the plane of shear to a further distance from the particle system and thus results in a reduction in the value of the measured zeta potential^20^,^21^. The UV-Vis spectra of AgNPs, Ag/PVA NPs and Ag/PEG NPs (Fig. 3A–D) showed a sharp Plasmon absorption peak at ~400 nm, which is the characteristic peak of spherical, mono-dispersed and well-dispersed AgNPs^22^,^23^. However, the UV-Vis spectrum of Ag/PVP NPs (Fig. 3D) showed a sharp peak at 420 nm. Previous studies demonstrated that spherical and mono-dispersed Ag/PVP NPs display a SPR band at ~412–437 nm^24^. The FT-IR spectra also confirmed the formation of AgNPs and core-shell Ag/polymeric (PVA, PEG, and PVP) NPs^24^,^25^,^26^,^27^ (Supplementary Figs S1–S4).

### Synthesis and Characterization of DOX-NCs

Following the synthesis of AgNPs and core-shell Ag/polymeric NPs, each individual type of NP was loaded with DOX. The drug loading efficiency was determined based on the DOX content in the supernatant. The drug loading efficiency percentages (Table 1) were determined to be: 58.3%, 54.9%, 56.5% and 62.5% for AgNPs, core-shell Ag/PVA NCs, core-shell Ag/PEG NCs and Ag/PVP NCs, respectively. The conjugation between DOX and NPs was detected using, TEM, SEM, and UV-Vis spectra (Fig. 3). The UV-Vis spectra (Fig. 3I–L) indicated that the binding between DOX and NPs resulted in a red shift of the Plasmon absorption band of loaded NCs from 400 to ~500 nm. The size and zeta potential of DOX-NCs (Table 1) were also measured and the results indicated that the size of NCs were not significantly increased as compared to their unloaded counterparts. The zeta potential values of the DOX-NCs (Table 1) were shifted to more negative values compared to their unloaded counterparts, and thus confirming the stability of the synthesized DOX-NCs. Previous studies demonstrated that the negatively charged NCs were beneficial for biomedical applications because they were slowly eliminated from the blood stream and had a lower cytotoxicity as compared to positively charged NCs^28^,^29^.

### *In-vitro* drug release

**Figure 4:**
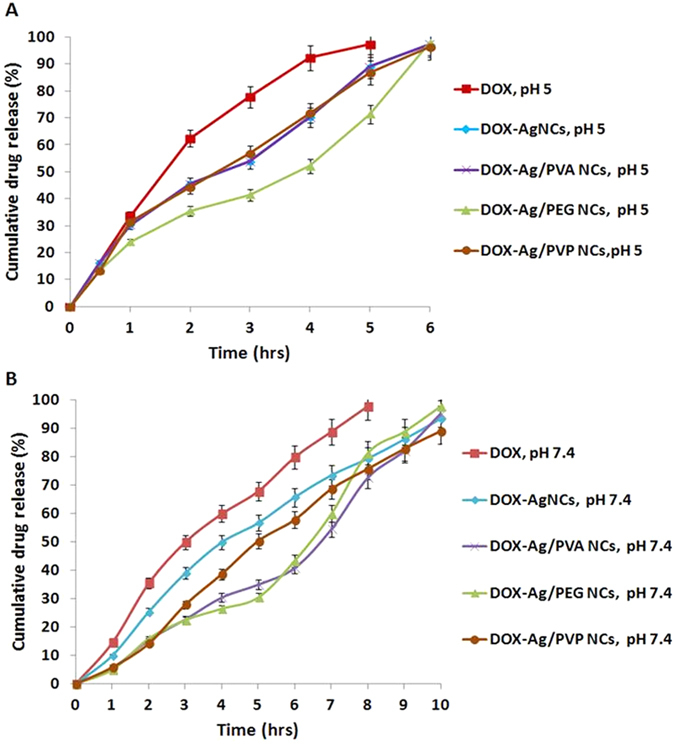
*In-vitro* release of free DOX, and DOX- NCs in Tris-HCl buffer pH 5 (**A**) and PBS pH 7.4 (**B**).

Since the release behavior of DOX-NCs at the desired site is of a great importance for formulating an ideal cancer-targeted drug delivery system, *in-vitro* release studies were performed. For this purpose, two different pH values were tested: pH 7.4, which mimics the pH of the blood stream and pH 5, which mimics the pH of the endosomes within cancer cells. *In-vitro* release study results (Fig. 4A,B) showed that DOX-AgNCs, DOX-Ag/PVA NCs, DOX-Ag/PEG NCs, and DOX-Ag/PVP NCs released 96.6%, 97.4%, 98% and 96.4% of DOX at pH 5. While at pH 7.4, the release percentages of DOX were 73.4%, 54.3%, 59.8% and 68.5% over the period of 6 hrs. On the other hand, free DOX solution was also used as a control and it was found that free DOX released 97.4% of DOX at pH 5, and 67.7% at pH 7.4 over the period of 4 hrs.

### *In-vitro* Cytotoxicity assay

#### Effect of AgNPs and Core-shell Ag/polymeric on MCF-7 cells and 1BR hTERT cells

**Figure 5:**
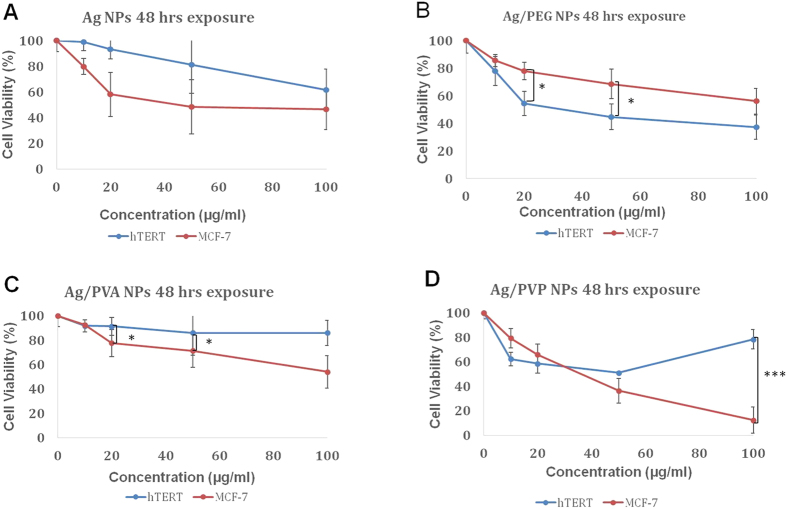
The percentage of viable MCF-7 cells (red) and 1BR hTERT cells (blue) as determined by the MTT assay following 48 hrs incubation with concentrations of 0, 10, 20, 50 and 100 μg/ml of: (**A**) AgNPs, (**B**) Ag/PVA NPs, (**C**) Ag/PEG NPs and (**D**) Ag/PVP NPs. The data are presented as means of at least three independent experiments (mean ± SD). P values were calculated for each concentration, and denoted if found to be significantly different between the two cell lines (*P < 0.05, **P < 0.01 and ***P < 0.001).

In order to assess the cytotoxic effect of AgNPs and core-shell Ag/polymeric NPs, MCF-7 and 1BR hTERT cells were exposed separately to different concentrations of (0, 10, 20, 50, and 100 μg/mL) NPs for 48 hrs. AgNPs and core-shell Ag/polymeric (PVA, PEG and PVP) NPs decreased the cell viability of MCF-7 cells and 1BR hTERT cells (Fig. 5A–D) in a dose-dependent manner. The inhibitory concentration (IC_50_) was estimated to be 48 μg/mL for AgNPs, 42 μg/mL for Ag/PVP NPs and greater than 100 μg/mL for both Ag/PVA NPs and Ag/PEG NPs on MCF-7 cells. The IC_50_ of NPs in 1BR hTERT cells was estimated to be 100 μg/mL for AgNPs, Ag/PVA NPs, and Ag/PEG NPs, while the IC_50_ of Ag/PVP NPs was 50 μg/mL. The Ag/PVA and Ag/PVP NPs were more cytotoxic against cancer cells at the high concentration of 100 μg/mL, with Ag/PVP NPs being more cytotoxic against MCF-7 cancer cells (Fig. 5A–D).

#### Effect of DOX-core-shell Ag/polymeric NPs on MCF-7 cells and 1BR hTERT cells

**Figure 6:**
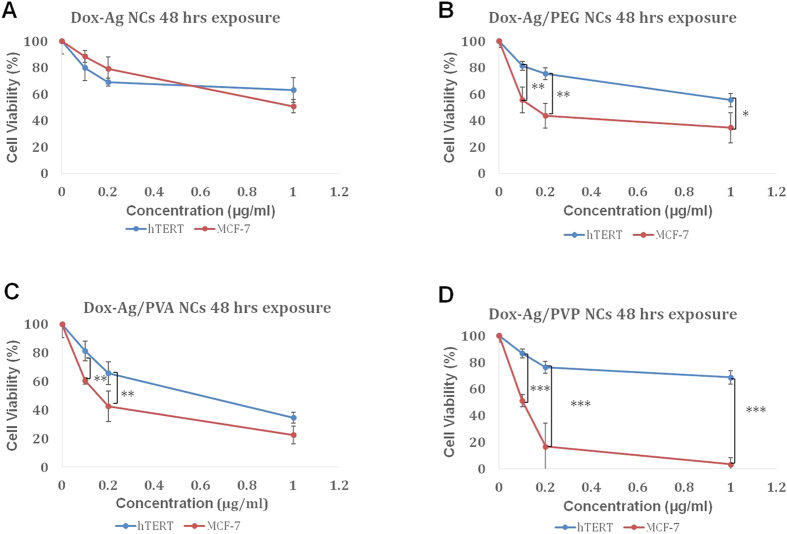
The percentage of viable MCF-7 cells (red) and 1BR hTERT cells (blue) as determined by the MTT assay following 48 hrs incubation with concentrations of 0, 0.1, 0.2 and 1 μg/ml (concentrations are referring to DOX concentration) of: (**A**) DOX-Ag NCs, (**B**) DOX-Ag/PVA NCs (**C**) DOX-Ag/PEG NCs, and (**D**) DOX-Ag/PVP NCs. The data are presented as means of at least three independent experiments (mean ± SD). P values were calculated for each concentration between the two cell lines, and denoted if found to be significant (*P < 0.05, **P < 0.01 and ***P < 0.001).

To investigate the cytotoxic effect of NPs-based combinatorial therapy, different concentrations of free DOX (2, 4, 8, 10, and 12 μg/mL) were tested on MCF-7 and 1BR hTERT cells, and cell viability was measured after 48 hrs. The IC_50_ of free DOX on MCF-7 cells was determined to be 3.7 μg/mL (Supplementary Fig. S5). Based on the IC_50_ of free DOX, lower DOX-NCs concentrations than the calculated IC_50_ of free DOX were selected (0.1, 0.2, and 1 μg/mL DOX) in order to assess whether the combination between DOX and NPs would induce synergism or not. The estimated IC_50_ values of DOX-NCs against both MCF-7 cells and 1BR hTERT cells, together with the individual concentrations of DOX and Ag in each DOX-NC -which lead to 50% cytotoxicity of both cell lines- are presented in Table 2. The estimated IC_50_ values (Table 2) of DOX-AgNCs, DOX-Ag/PVA NCs, DOX-Ag/PEG NCs and DOX-Ag/PVP NCs against MCF-7 cells were 1.00–11.23 μg/mL, 0.19–3.40 μg/mL, 0.14–3.00 μg/mL, and 0.10–3.50 μg/mL, respectively (Fig. 6A–D). On the other hand, the estimated IC_50_ values (Table 2) of DOX-NCs against 1BR hTERT cells were 1.00–11.23 μg/mL for DOX-AgNCs, DOX-Ag/PEG NCs, and DOX-Ag/PVP NCs, while the estimated IC_50_ value of DOX-Ag/PVA NCs was 0.60–9.00 μg/mL (Fig. 6A–D). All DOX-loaded core-shell Ag/polymeric NCs were found to be more cytotoxic against cancer cells versus normal cells. Notably, the Dox-Ag/PVP combination was more cytotoxic than all three and was more cytotoxic on cancer cells.

## Discussion

In other studies DOX was loaded to different carriers such as liposomes, polymeric NPs, carbon nanotubes, however, few studies focused on formulating a combination therapy based on using DOX and AgNPs. In this study, DOX was loaded to AgNPs and core-shell Ag/polymeric NPs. DOX loading was confirmed by UV-visible spectroscopy. The UV-Vis spectra of DOX-NCs (Fig. 3I–L) showed a red shift - consistent with previously published data^30^,^31^, which probably resulted from a change in pH due to the interaction of the NPs with DOX –which in itself is of acidic nature. The red shift of UV-Vis spectra was also attributed to the binding between DOX and AgNPs, which resulted in the decrease in inter-particle distance of NPs. Though the red shift of UV-Vis spectra and low values of Zeta potential both suggest that there are some NPs aggregations, however, the TEM and SEM images of DOX-NCs (Fig. 3) confirmed that the DOX-NCs remained well-dispersed. A similar observation on the well-dispersity of the NCs after their binding with DOX has also been reported by Kumar *et al.*^30^. Therefore, by combining the results obtained from the UV-Vis spectra, the TEM images, as well as the SEM images of unloaded-NPs and DOX-NCs, it can be inferred that DOX and NPs were successfully binding to each other while maintaining the well-dispersity of the NCs.

The *in-vitro* release studies (Fig. 4A,B) also demonstrated that free DOX was released faster than DOX-NCs at both of the tested pH values. The delay of DOX release from DOX-NCs was due to the binding of DOX with the different NPs, which accordingly improved the release profile of DOX and prolonged its half-life compared to free DOX. Moreover, the results confirmed that both DOX-NCs and free DOX exhibited faster release in pH 5, which mimics the pH of endosomes within cancer cells, when compared to their release in pH 7.4. The fast release of free DOX was based on the fact that protonated DOX has a higher solubility. However, the fast release of DOX from DOX-NCs was due to weakened interaction between DOX and NCs at acidic pH. This is due to the protonation of DOX amino groups, which leads to DOX detachment from the NPs^31^,^32^,^33^. The pH-sensitivity property of DOX-AgNCs complexes seems to be advantageous for cancer-targeted drug delivery because the acidic microenvironment of cancer cells facilitates active drug release from NCs, increases drug bioavailability to cancer cells, and leads to high therapeutic efficacy compared to normal cells.

The cytotoxicity of unloaded AgNPs and core-shell Ag/polymeric NPs were examined on MCF-7 cells and 1BR hTERT cells by the MTT assay. AgNPs and core-shell Ag/polymeric NCs –except Ag/PEG NPs-showed higher cytotoxicity on cancer MCF-7 cells compared to normal 1BR hTERT cells (Figs 5 and 6). These results imply that AgNPs particularly coated with PVP, and to a lesser extent coated with PVA, are specifically cytotoxic to MCF-7 cancer cells, when compared to normal 1BR hTERT cells. This is in contrast to the PEG coating, which resulted in more cytotoxicity to normal cells. Based on previous studies, the cytotoxic effect of AgNPs was ascribed to their ability to dissolve and release Ag^+^ ions, which have a great potential to translocate to both the mitochondria and nucleus, thereby triggering the generation of ROS and mediating oxidation stress. The oxidation stress causes a series of cellular events including the reduction of glutathione (GSH) and superoxide dismutase (SOD) levels as well as increasing lipid peroxidation, which finally lead to DNA damage and cancer cell death^14^,^15^,^16^. The difference in the estimated IC_50_ values among NPs is probably attributed to the different surface coating among NPs. Previous studies documented that the surface coating of AgNPs controls their dissolution and influences their cytotoxicity^33^,^34^,^35^. In concordance, this study confirmed that the surface coating of NPs directly influences their cytotoxic effects. MTT assay results showed that Ag/PVP NPs exhibited the highest cytotoxicity (IC_50_: 42 μg/mL) as compared to AgNPs (IC_50_: 48 μg/mL), Ag/PVA NPs and Ag/PEG NPs (IC_50_: above 100 μg/mL) (Fig. 5). Dobias and Bernier-Latmani reported that core-shell Ag/PVP NPs exhibited a higher cytotoxic effect than AgNPs because of its intrinsic higher dissolution than AgNPs^36^,^37^,^38^,^39^,^40^. However, Luo *et al.* revealed that core-shell Ag/PEG NPs exhibited slow dissolution due to the binding of detached negatively charged PEG polymer chains with released Ag^+^ ions forming stable Ag-ligand complexes resulting in Ag^+^ ions retention and decreased cytotoxicity^38^.

To verify the combined effect of DOX and Ag on MCF-7 cells, the cytotoxicity of free DOX and DOX-NCs were examined on MCF-7 cells and 1BR hTERT cells by the MTT assay (Fig. 6). DOX-NCs possess an enhanced inhibitory effect on MCF-7 cells at very low doses when compared to 1BR hTERT cells. In fact the coated DOX-AgNCs were highly cytotoxic to MCF-7 cancer cells when compared to the normal cell counterpart-1BR hTERT. This implies that loading coated AgNPs with DOX renders DOX AgNPs more selective to cancer cells; particularly PVP coating that induced the highest cytotoxicity to cancer cells-based on the significant difference in cytotoxicity between normal and cancer cells (Fig. 6D). MTT results on MCF-7 cells revealed that core-shell Ag/polymeric NCs showed a 10-fold reduced DOX IC_50_ compared to free DOX and DOX-AgNCs. These results confirmed that a synergistic anti-cancer effect is induced by DOX-NCs, which could be possibly ascribed to: (i) the combined cytotoxic effect of AgNPs with the therapeutic effect of DOX and (ii) the enriched internalization of DOX-NCs NCs, via endocytosis, allowing the release of DOX inside the cell as compared to the passive diffusion of free DOX into the cells. It was previously reported that nanocarriers mediate endocytosis, leading to an enhancement in cellular internalization. Venkatpurwar *et al.* reported a significant enhancement in the cytotoxicity of DOX-AuNCs on human glioma cell line (LN-229) compared to free DOX, possibly through enhanced cellular internalization owing to AuNPs mediated endocytosis^40^. Chen *et al.* also reported the passive intracellular accumulation of methotrexate-AuNCs, confirming AuNCs mediated endocytosis followed by methotrexate release inside cancer cells^41^. Further molecular assays should be conducted in the future in order to determine experimentally the mechanism of action underlying cancer cell death after exposure to DOX-AgNCs; whether the cells died by apoptosis or other cellular death pathways.

Finally, this study confirmed that combining DOX and core-shell Ag/polymeric (PVA, PEG, and PVP) NPs at very low doses possess a synergic cytotoxic effect on MCF-7 cells. The inhibitory concentrations of DOX-NCs against cancer cells do not cause a significant decrease in the viability of normal cells and this highlights the preliminary importance of AgNPs in breast cancer chemotherapy. Additionally, combining much lower doses of DOX and core-shell Ag/polymeric NPs could aid in formulating novel targeted cancer nanotherapeutics possessing synergic anti-cancer effect while possibly minimizing the adverse side effects.

## Conclusion

Mono-dispersed spherical AgNPs and core-shell Ag/polymeric (PVA, PEG, and PVP) NPs were successfully synthesized, loaded with DOX, and the *in-vitro* drug release of each individual type of DOX-NCs was investigated. Moreover, an individual unloaded-NP, free DOX and DOX-NC were tested for *in-vitro* cytotoxicity on MCF-7 cells and 1BR hTERT cells. *In-vitro* MTT assay results demonstrated that core-shell DOX-Ag/polymeric NCs at much lower doses- showed a synergic cytotoxic effect towards MCF-7 cells, and a lower cytotoxic effect on normal 1BR hTERT cells. Finally, to complement and confirm the synergism and overall efficacy of DOX-Ag/polymeric NCs, several studies such as *in-vitro* and *in-vivo* toxicity studies and *in-vivo* anti-tumor activity on cancer cells and normal cells are recommended. These further studies could progress the proposed NPs-based combinational therapeutic to formulate a novel targeted cancer therapy that could be used in clinical trials; as it can potentially eradicate cancer cells selectively and effectively while minimizing the adverse side effects.

## Methods

### Preparation of AgNPs and core-shell NPs

AgNPs, core-shell Ag/PVA NPs and Ag/PEG NPs were prepared by chemical reduction method with some modification as reported previously^24^,^25^. Core-shell Ag/PVP NPs were synthesized by polyol process^24^. All preparation methods were described in the supplementary information.

### Synthesis of DOX-AgNCs

Briefly, 1 mL aqueous solution of DOX (0.2 mg/mL) was mixed with an aqueous solution (2 mg/mL) of each individual type of AgNPs at pH 7.4. Each mixture was shaken in a rotary shaker at 37 °C for 24 hrs in dark conditions. Then, the mixtures were centrifuged for 15 min and the supernatants were used to determine the amount of free DOX by UV-Vis spectroscopy at 480 nm^30^. The drug loading efficiency was calculated as follows^30^:


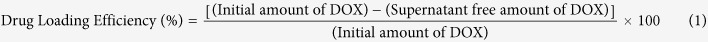


### *In-vitro* drug release

1 mL of individual DOX-NC was dispersed in de-ionized water and then transferred into a dialysis bag (cut off molecular weight 12,000–14,000 g/mol, Serva, Germany) containing 50 mL PBS buffer (pH 7.4) and Tris-HCl buffer (pH 5) respectively, with the temperature maintained at 37 °C. At fixed time intervals, 1 mL of the medium was withdrawn from each dialysis bag and subsequently replaced with fresh buffer to maintain the sink conditions^30^. The amount of released DOX was determined by UV-Vis spectroscopy at 480 nm. The cumulative percentage of drug release was calculated as follows^30^:


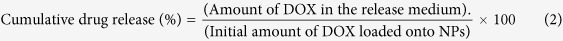


### Cytotoxicity assay

The effects of different NPs were tested on two distinct, yet conventional cell lines; wild-type human telomerase reverse transcriptase immortalized cell lines-hTERT cells-1BR skin fibroblast (1BR hTERT cells) and the human breast adenocarcinoma cell line (MCF-7)^42^. 1BR hTERT cells, were a gift of Dr. Andreas Kakarougkas (University of Sussex), were used as control cells; as they are non-cancerous –normal- immortalized human skin fibroblasts^43^,^44^,^45^. The cytotoxicity experiments aimed to assess and compare the cytotoxicity of the different studied NPs on breast cancer cells –MCF-7 cells- and normal fibroblasts -1BR hTERT cells. The cytotoxic effect of the aforementioned concentrations of DOX, AgNPs, core-shell Ag/polymeric NPs, and the DOX-NCs on MCF-7 cells and 1BR hTERT fibroblasts were conducted by the 3-(4, 5-dimethylthiazolyl-2)-2, 5-diphenyltetrazolium bromide (MTT) assay^27^,^32^. The absorbance (A) was measured using FLUOstar OPTIMA microplate reader (BMG LabTech, Germany) at 595 nm. The percentage of cell viability was calculated as follows^32^,^34^:


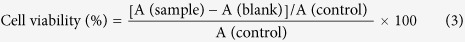


For the IC_50_ values (Table 2), the Dox concentrations were calculated from the cytotoxicity assay data using GraphPad Prism version 6.00 for Windows, GraphPad Software, La Jolla California USA, www.graphpad.com. For the corresponding Ag IC_50_ values, they were calculated in relevance to Ag and by the DOX loading efficiency (equation 1)^27^.

### Statistical analysis

All the cell viability percentage values (Figs 5 and 6) were analyzed by Tukey’s HSD statistical test one-way ANOVA pair-wise comparisons by using version 2.14.1.

## Additional Information

**How to cite this article**: Elbaz, N. M. *et al.* Core-Shell Silver/Polymeric Nanoparticles-Based Combinatorial Therapy against Breast Cancer *In-vitro. Sci. Rep.* **6**, 30729; doi: 10.1038/srep30729 (2016).

## Supplementary Material

Supplementary Information

## Figures and Tables

**Figure 1:**
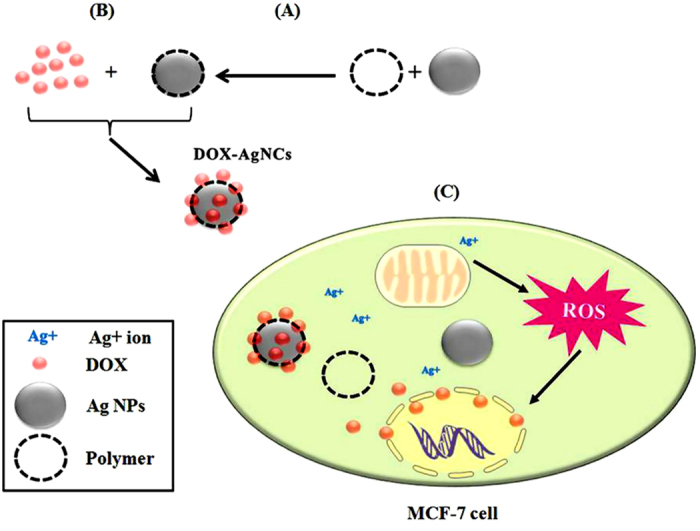
Schematic illustration of the study workflow and the possible mechanism of action behind the resultant synergic cytotoxic effect of DOX-loaded Ag/polymeric NCs at very low doses of DOX on MCF-7 cells. (**A**) Preparation of NPs, (**B**) DOX loading and (**C**) Ag^+^ ions and DOX intracellular release possibly leads to mitochondrial dysfunction that generates ROS leading to DNA fragmentation and cell death.

**Table 1:** Composition, Size, Zeta potential, and drug loading of prepared NPs and DOX NCs.

Sample Code	Composition			Meandiameter	PDI	Zeta	LE
	**Ag (mg)**	**Capping agent (mg)**	**DOX (mg)**	**(nm)**		**(mV)**	**%**
AgNPs	100	250	—	14.48 ± 1.21	0.27 ± 0.02	−12.43 ± 1.20	
DOX-AgNPs	2	5	0.2	22.21 ± 4.75	0.33 ± 0.05	−20.83 ± 0.14	58.3
Ag/PVA NPs	100	250	—	18.59 ± 2.91	0.41 ± 0.03	−0.30 ± 0.50	
DOX-Ag/PVA NCs	2	5	0.2	24.28 ± 3.93	0.26 ± 0.06	−6.72 ± 0.90	54.9
Ag/PEG NPs	100	250	—	20.16 ± 4.15	0.14 ± 0.02	−2.35 ± 1.76	
DOX-Ag/PEG NCs	2	5	0.2	23.85 ± 1.73	0.36 ± 0.05	−5.78 ± 1.07	56.5
Ag/PVP NPs	10	50	—	21.56 ± 2.10	0.31 ± 0.04	−12.4 ± 1.20	
DOX-Ag/PVP NCs	2	5	0.2	28.54 ± 1.18	0.22 ± 0.08	−16.89 ± 1.53	62.5

**Table 2:** Estimated IC_50_ values of DOX-NCs against both MCF-7 cells and 1BR hTERT cells.

Formulae	IC_50_			
	MCF-7 cells		1BR hTERT cells	
	DOX (μg/mL)	Ag (μg/mL)	DOX (μg/mL)	Ag (μg/mL)
DOX-AgNCs	1.00	11.23	1.00	11.23
DOX-Ag/PVA NCs	0.19	3.40	0.60	9.00
DOX-Ag/PEG NCs	0.14	3.00	1.00	11.23
DOX-Ag/PVP NCs	0.10	3.50	1.00	11.23

Individual concentration of DOX and Ag in each DOX-NC leading to 50% cytotoxicity of both cell lines are presented.
