# Supplementary material for: Core-Shell Silver/Polymeric Nanoparticles-Based Combinatorial Therapy against Breast Cancer In-vitro
Source: Sci Rep. 2016 Aug 5;6:30729. doi: 10.1038/srep30729 (PMC4974507; doi:10.1038/srep30729)
Supplement: Supplementary Information [file srep30729-s1.pdf]

Supplementary Information

Core-Shell Silver/Polymeric Nanoparticles-Based Combinatorial Therapy against Breast Cancer *In-vitro*

Nancy M. El-Baz[1,2] , Laila Ziko[1,3], Rania Siam[1,3], Wael Mamdouh[1,2]*

[1]Yousef Jameel Science and Technology Research Center (YJSTRC), School of Sciences and Engineering, American University in Cairo, 11835, Egypt

[2]Department of Chemistry, School of Sciences and Engineering, American University in Cairo, 11835, Egypt

[3]Department of Biology and Biotechnology Graduate Program, School of Sciences and Engineering, American University in Cairo, 11835, Egypt

Methods:

1.1. Synthesis of AgNPs

AgNPs were synthesized using modified procedures to Kang *et al.* study [23]. Briefly, 2 ml of an aqueous solution of silver nitrate was added to 48 ml of 1% tri-sodium citrate and mixed under vigorous stirring for 15 min in dark conditions. 2 mM sodium borohydride solution was afterwards added drop-wise until the color of the solution changed into yellow and then centrifuged at 12,000 rpm for 15 min. Finally the AgNPs were re-dispersed in de-ionized water and stored in the dark at 4 ºC.

1.2. Preparation of core-shell Ag/PVA NPs and Ag/ PEG NPs

Core-shell Ag/PVA NPs and Ag/PEG NPs were prepared by chemical reduction method with some modification as reported previously [24]. Briefly, 2 ml of silver nitrate solution was added to 48 ml of 1% polymer and was stirred for 15 min under dark conditions. 2 mM of sodium borohydride was then added drop-wise until the solution color turned into yellow. Afterwards, the solutions were centrifuged at 12,000 rpm for 15 min. and finally the NPs were re-dispersed in de-ionized water and stored in the dark at 4 ºC.

1.3. Preparation of core-shell Ag/PVP NPs

Core-shell Ag/PVP NPs were synthesized by the polyol process [25]. Briefly, 0.001 M of PVP was dissolved in ethylene glycol and heated up to 180 ºC and then the temperature was lowered and maintained at 120 ºC. Then, 0.1 g silver nitrate solution was added to the above solution and stirred for 3 min under dark conditions and then left to cool at room temperature. Afterwards, excess amount of acetone was added allowing the formation of a brown precipitate. Finally, the solution was centrifuged at 12,000 rpm for 15 min and then the NPs were re-dispersed in de-ionized water and stored in the dark at 4 ºC.

Characterization:

The morphological characteristics of AgNPs, core-shell NPs and DOX-loaded NPs were examined by a Scanning Electron Microscope (FESEM, Leo Supra 55 – Zeiss Inc., Germany). The SEM samples were prepared by dropping the solution onto the foil-coated copper grid and subsequently drying in air. The morphology of samples was also determined using high-performance digital imaging transmission electron microscope (TEM) (JEM-2100F; JEOL, USA). One drop of the nanoparticle suspension was stained and spread onto a carbon-coated copper grid. The sample was left for drying at room temperature. After drying, the samples were placed for TEM analysis with 100 kV accelerating voltage. The composition of AgNPs and core-shell Ag/polymeric NPs was confirmed using infrared spectrometer (FT-

IR) (Nicolet 380-Thermo Scientific, USA). The spectra were obtained in the range of 500 and 4000 cm−1. The absorption bands of AgNPs, core-shell Ag/polymeric NPs and DOX-loaded NPs were measured by Ultraviolet–Visible (UV-Visible) spectroscopy (CARY 500, Agilent Technologies, USA). The UV-Vis spectra were obtained with a 1-cm quartz cell in the wavelength ranged between 300-700 nm. The measurements of particle diameter, PDI and Zeta-potential of void and loaded void NPs and loaded NPs were performed at 25◦C using a Zetasizer (Nano-ZS; Malvern Instruments, Malvern, UK) on the basis of dynamic light scattering (DLS) and laser Doppler electrophoresis.

FTIR measurement:

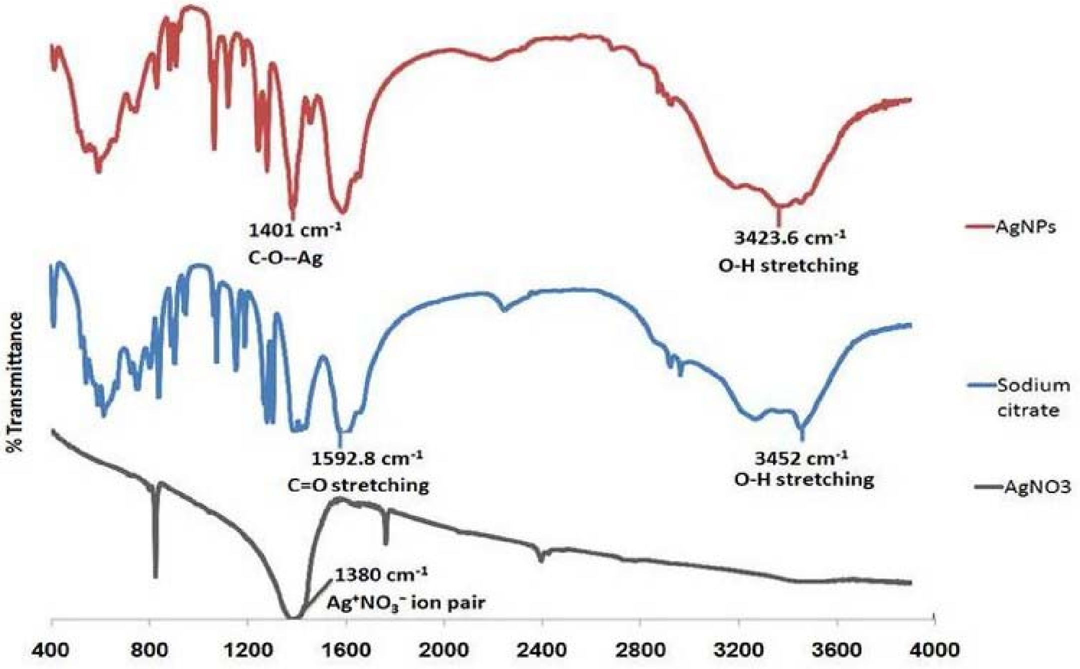

Figure S1: FTIR spectra of silver nitrate, sodium citrate, and AgNPs.

In addition, the FT-IR spectra of AgNPs, trisodium citrate and $AgNO_3$ in confirm the formation of AgNPs. By comparing the FT-IR spectra of AgNPs, sodium citrate and $AgNO_3$, the FT-IR spectra of AgNPs showed a band at 3423.6 cm^{-1} that is not present in the FT-IR spectrum of $AgNO_3$. This band is attributed to O-H stretching between AgNPs and trisodium citrate. In addition, it was found that the band at 1592.8 cm^{-1} is assigned for symmetric carboxylic group stretching mode of sodium citrate underwent a blue shift and appeared sharply at 1401 cm^{-1} in the spectrum of AgNPs and thus confirming the stabilization of AgNPs by carboxylic group of

trisodium citrate. Moreover, the spectrum of $AgNO_3$ displays a band at 1380 cm^{-1} corresponding to ion pair $Ag^+NO_3^-$ that is not found in the spectrum of AgNPs due to the separation of NO_3^- from its Ag^+ counterpart.

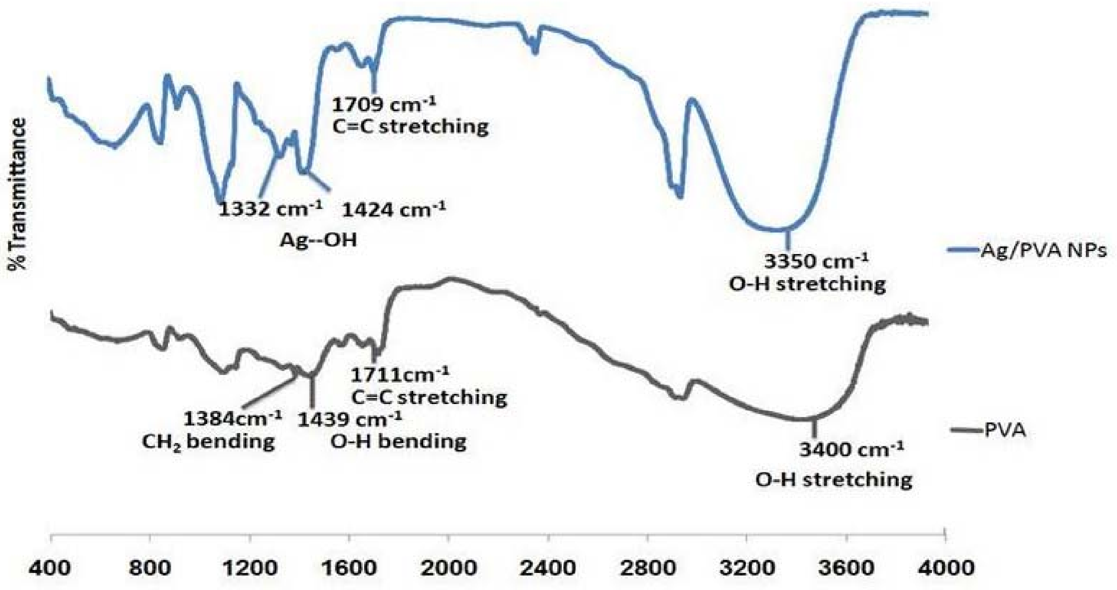

Figure S2: FTIR spectra of Pure PVA and core-shell Ag/PVA NPs.

The FT-IR spectra of pure PVA and core-shell Ag/PVA NPs are found to be nearly similar. Both spectra show a broad band at 3400 cm^{-1} for pure PVA and 3350 cm^{-1} for core-shell Ag/PVA NPs respectively, which corresponds to the stretching vibration of hydroxyl group of PVA. Both FT-IR spectra show a band at 2942 cm^{-1} is assigned to CH_2 asymmetric stretching vibration. The bands at 1711cm^{-1} for pure PVA and 1709 cm^{-1} for core-shell Ag/PVA NPs corresponds to C=C stretching mode. In addition, the band at 1655cm-^{1} assigned to C=O group which could be contributed to the intra/ inter molecular hydrogen bond with adjacent hydroxyl group. Moreover, both FT-IR spectra displayed a band at 1095 cm^{-1} for pure PVA and 1093 cm^{-1} for core-shell Ag/ PVA NPs which are assigned to C-O stretching of acetyl group on PVA back bone. Additionally, the band at 1439 cm^{-1} and 1384cm^{-1} for pure PVA may be attributed to CH_2 and O-H bending

vibrations. However, these bands underwent a blue shift and appeared at 1424 cm^{-1} and 1332 cm^{-1} in the spectrum of core-shell Ag/PVA NPs. This blue shift of the bands observed at 1424cm^{-1} indicates that PVA polymer is adsorbed on the surface of AgNPs via the interaction of AgNPs and OH groups of PVA.

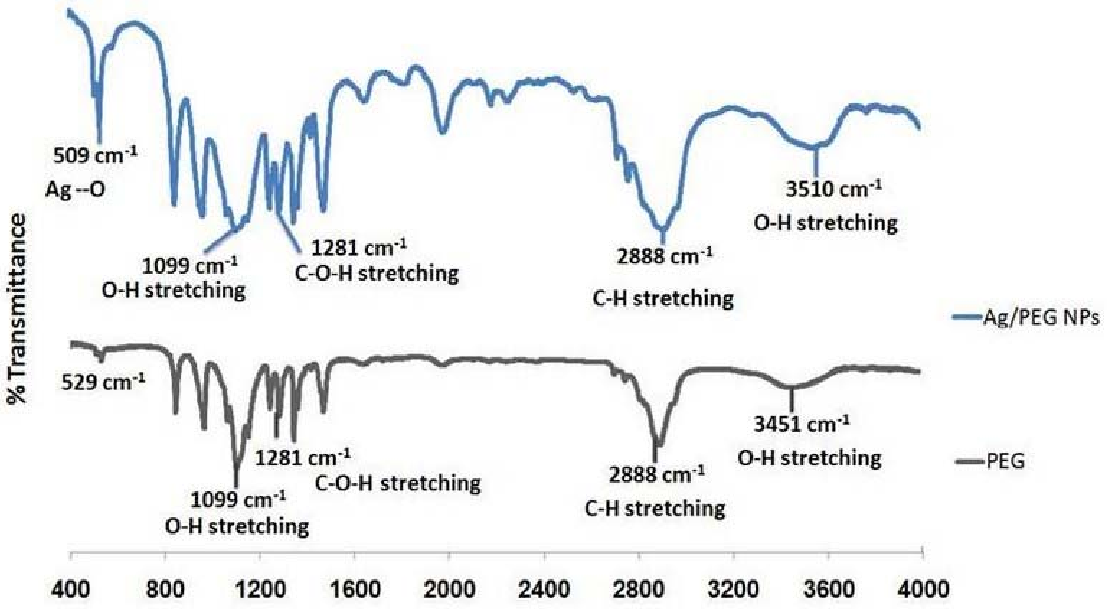

Figure S3: FTIR spectra of Pure PEG and core-shell Ag/PEG NPs.

The FT-IR spectra of pure PEG and core-shell Ag/PEG NPs, also confirmed the formation of core-shell Ag/PEG NPs. The FT-IR spectra of pure PEG and core-shell Ag/PEG NPs are nearly identical. The FT-IR spectrum of pure PEG shows a band at 3451 cm^{-1} corresponds to O-H stretching vibrations and a band at 2888 cm^{-1} corresponds to C-H stretching vibrations. Both FT-IR spectra showed bands at 1466 and 1342 cm^{-1} which are assigned to C-H bending vibrations. The stretching vibrations of alcoholic OH and C-O-H stretching are observed at 1281 and 1099 cm^{-1} also appear in both spectra. However, the FT-IR spectrum of Ag/PEG NPs showed a broad band at 1099 cm^{-1} as compared to FT-IR spectrum of PEG, which could be ascribed to C-O-H vibrations of AgNPs in PEG. Comparing the FT-IR of both pure PEG and the spectrum of core-shell Ag/ PEG NPs, a strong band is observed at 509 cm^{-1} at the spectrum of core-shell Ag/PEG NPs, which could be attributed to AgNPs banding with oxygen from hydroxyl groups of PEG

chains and thus suggesting the existence of van der Waals interaction between the positively charged groups on the surface of Ag NPs and the negatively charged oxygen from the hydroxyl groups of PEG.

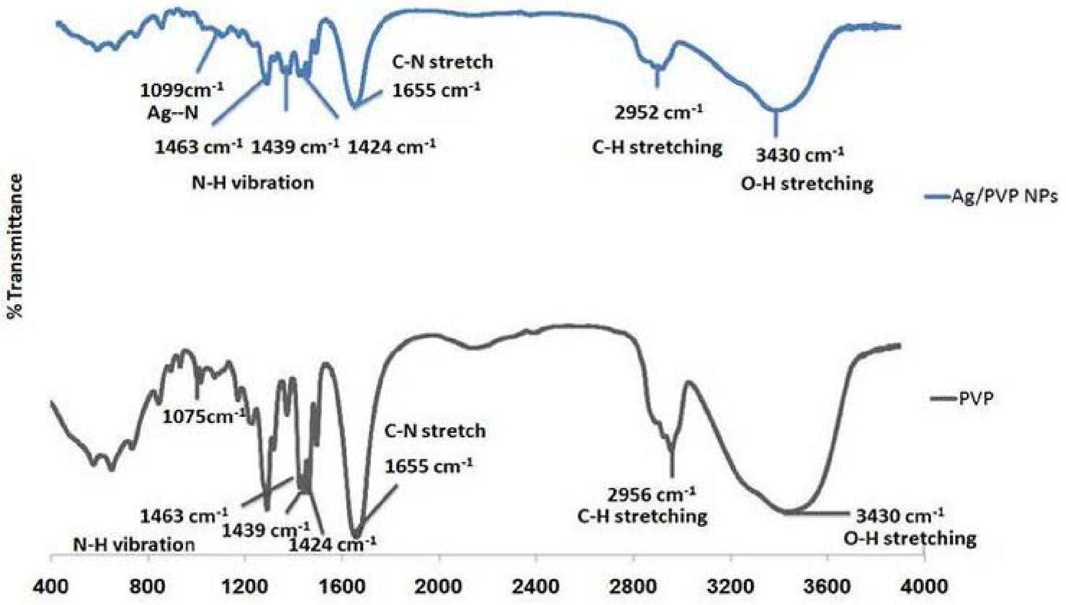

Figure S4: FTIR spectra of Pure PVP and core-shell Ag/PVP NPs.

The FT-IR spectra of pure PVP and core-shell Ag/PVP NPs formed by polyol method shown are closely identical. Both spectra showed peaks at 3430 cm^{-1} corresponds to O-H stretching vibration of hydroxyl group and 2952 cm^{-1} corresponding to symmetric stretching vibration of C-H bond. Both spectra also showed a sharp band at 1655 cm^{-1} that corresponds to amide carbonyl stretch absorption and peaks at 1463, 1439, 1424 cm^{-1} are assigned to the vibration of tertiary nitrogen. However, the FT-IR spectrum of core-shell Ag/PVP NPs displays a red shift at band 1099 cm^{-1}, compared to the band at 1075cm^{-1} in the spectrum of pure PVP. The red shift of band at 1099cm^{-1} confirms the involvement of pyrrolidyl nitrogen electrons in the formation of core-shell Ag/PVP NPs. This emphasizes that PVP is adsorbed at the silver NPs surfaces through donating electrons from the N atom to the Ag or the coordination between N and Ag. Wang *et al.* revealed that core-shell Ag/PVP NPs with diameters smaller than 50 nm are protected by PVP via a coordination bond between N in PVP and Ag as shown in while NPs with diameters greater than 50nm both N and O in PVP coordinated with Ag.

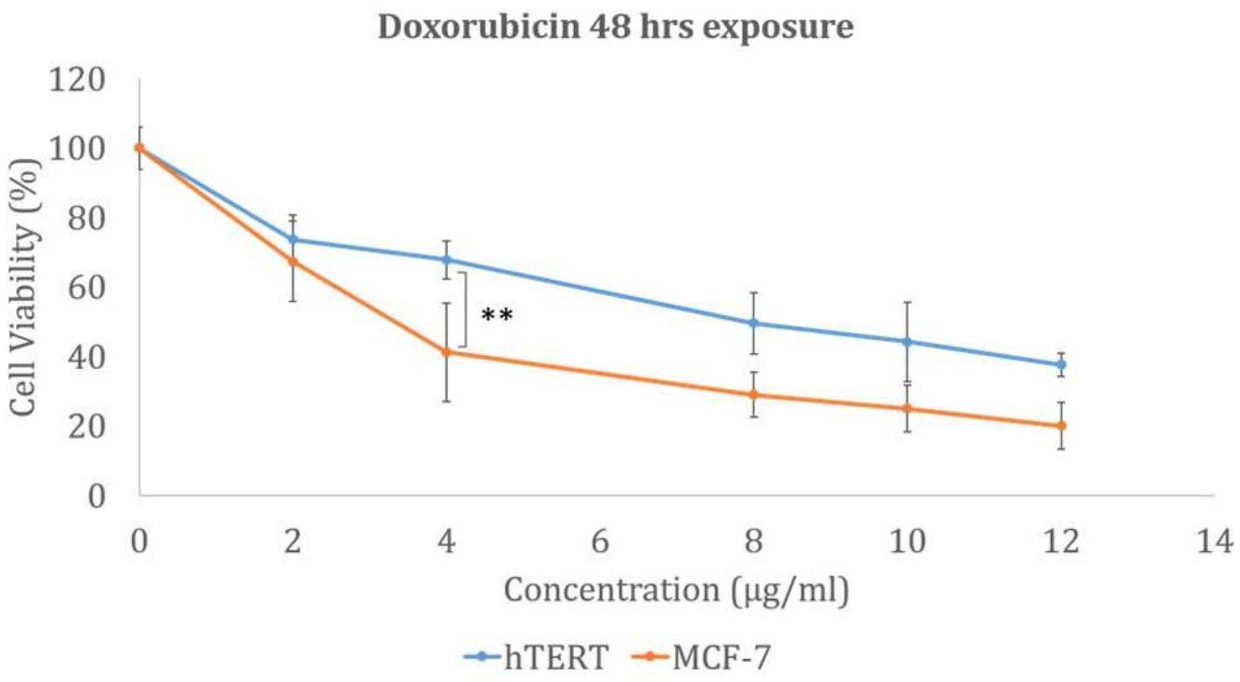

Figure S5: **:** The percentage of viable MCF-7 cells (red) and 1BR hTERT cells (blue), after exposure to DOX as determined by the MTT assay following 48 hrs incubation with concentrations of 0, 2, 4, 8, 10 and 12µg/ml. The data are presented as a mean of at least three independent experiments (mean ± SD). P values were calculated for each concentration between the two cell lines, and denoted if found to be significant (*P < 0.05, **P < 0.01 and ***P < 0.001).
